# Cognitive impairment in Parkinson’s disease: a report from a multidisciplinary symposium on unmet needs and future directions to maintain cognitive health

**DOI:** 10.1038/s41531-018-0055-3

**Published:** 2018-06-26

**Authors:** Jennifer G. Goldman, Beth A. Vernaleo, Richard Camicioli, Nabila Dahodwala, Roseanne D. Dobkin, Terry Ellis, James E. Galvin, Connie Marras, Jerri Edwards, Julie Fields, Robyn Golden, Jason Karlawish, Bonnie Levin, Lisa Shulman, Glenn Smith, Christine Tangney, Cathi A. Thomas, Alexander I. Tröster, Ergun Y. Uc, Noreen Coyan, Crystal Ellman, Mike Ellman, Charlie Hoffman, Susan Hoffman, Don Simmonds

**Affiliations:** 1 Department of Neurological Sciences, Section of Parkinson Disease and Movement Disorders, Rush University Medical Center, Chicago, IL USA; 2 Parkinson’s Foundation, New York, NY USA; 3 Department of Medicine, University of Alberta, Edmonton, AB Canada; 4 Department of Neurology, University of Pennsylvania, Philadelphia, PA USA; 5 Department of Psychiatry, Rutgers The State University of New Jersey, New Brunswick, NJ USA; 6 Department of Physical Therapy and Athletic Training and Center for Neurorehabilitation, Boston University, Boston, MA USA; 7 Charles E. Schmidt College of Medicine, Florida Atlantic University, Boca Raton, FL USA; 8 Morton and Gloria Shulman Movement Disorders Centre and the Edmond J. Safra Program in Parkinson’s disease, Toronto Western Hospital, University Health Network, University of Toronto, Toronto, ON Canada; 9 University of South Florida, Tampa, FL USA; 10 Department of Psychiatry and Psychology, Mayo Clinic, Rochester, MN USA; 11 Department of Health and Aging, Rush University Medical Center, Chicago, IL USA; 12 Department of Medicine, University of Pennsylvania, Philadelphia, PA USA; 13 Department of Neurology, Division of Neuropsychology, University of Miami, Miami, FL USA; 14 Department of Neurology, University of Maryland School of Medicine, Baltimore, MD USA; 15 Department of Clinical and Health Psychology, University of Florida, Gainesville, FL USA; 16 Department of Clinical Nutrition, College of Health Sciences, Rush University Medical Center, Chicago, IL USA; 17 Department of Neurology, Boston University Medical Campus, Boston, MA USA; 18 Department of Clinical Neuropsychology and Center for Neuromodulation, Barrow Neurological Institute, Phoenix, AZ USA; 19 Department of Neurology, University of Iowa, and Neurology Service, Iowa City, Veterans Affairs Health Care Systen, Iowa City, IA USA; 20 Illinois, USA; 21 Colorado, USA

## Abstract

People with Parkinson’s disease (PD) and their care partners frequently report cognitive decline as one of their greatest concerns. Mild cognitive impairment affects approximately 20–50% of people with PD, and longitudinal studies reveal dementia in up to 80% of PD. Through the Parkinson’s Disease Foundation Community Choice Research Award Program, the PD community identified maintaining cognitive function as one of their major unmet needs. In response, a working group of experts across multiple disciplines was organized to evaluate the unmet needs, current challenges, and future opportunities related to cognitive impairment in PD. Specific conference goals included defining the current state in the field and gaps regarding cognitive issues in PD from patient, care partner, and healthcare professional viewpoints; discussing non-pharmacological interventions to help maintain cognitive function; forming recommendations for what people with PD can do at all disease stages to maintain cognitive health; and proposing ideas for how healthcare professionals can approach cognitive changes in PD. This paper summarizes the discussions of the conference, first by addressing what is currently known about cognitive dysfunction in PD and discussing several non-pharmacological interventions that are often suggested to people with PD. Second, based on the conference discussions, we provide considerations for people with PD for maintaining cognitive health and for healthcare professionals and care partners when working with people with PD experiencing cognitive impairment. Furthermore, we highlight key issues and knowledge gaps that need to be addressed in order to advance research in cognition in PD and improve clinical care.

## Introduction

### Perspectives on cognitive impairment from people with Parkinson’s disease and care partners in the working group

Quotes from people with PD:

“Sometimes my brain ‘freezes up,’ kind of like my legs sometimes do. Finding the words I want to say is very hard, and my thoughts seem like they are blank.”

“I wish that the doctor had told me that cognitive changes could be one of the results of the disease. My clinic visits have focused much more on physical signs and symptoms. The few times cognition has been addressed, the comments were very brief.”

Quotes from PD care partners:

“The most bothersome is attention, which we call ‘chasing rabbits’ at our house. Early on this manifested as flitting from task to task without completion. However, cognitive symptoms have started to affect communication, which in turn affects our relationship. My husband’s frustration in turn sparks irritation in both of us. I sometimes wonder if the same is not occurring with friends, and they are too polite to tell me.”

“My husband has lost his initiative in social situations. He listens but rarely contributes and may get confused in the conversation. I miss the social interactions that we used to have with friends with little planning. With just a suggestion, it used to happen.”

Along with people with PD and their care partners, a multidisciplinary group of experts convened for a conference workshop entitled, “Non-pharmaceutical ways to maintain cognitive function in PD.” This workshop stemmed from the 2016 Parkinson’s Disease Foundation Community Choice Research Award Program, in which cognitive function in PD was selected as a community priority. In response, a program on this topic was designed, bringing together clinicians and researchers with a wide range of expertise as well as people directly affected by PD. In attendance were 19 clinicians and researchers including: five movement disorder neurologists, one cognitive neurologist, two psychologists, four neuropsychologists, one physical therapist, one nutritionist, one registered nurse, one social worker, one geriatric neurologist, one gerontologist/clinical ethicist, and one researcher from the Parkinson’s Foundation. Also in attendance were three people with PD (one female, two male) and their care partner spouses (one male, two female). The conference covered nine topics, which were selected ahead of time by the conference leaders (J.G.G., B.A.V.): 1. defining cognitive issues in PD, 2. features and etiology of PD cognitive impairment, 3. cognitive aging, 4. clinical measures of cognition, 5. physical exercise, 6. cognitive exercise, 7. nutrition, 8. caregiver involvement in PD cognitive issues, and 9. decision-making capacity. Each topic was discussed with respect to its current state in the field, perceived unmet needs, and potential future directions, as based on literature review, expert opinion, and conference group discussion. Participants then generated questions and provided feedback after the conference, from which proposed recommendations were developed to help people with PD be proactive about their cognitive health. These questions aimed to elicit recommendations from healthcare professionals working with PD patients across disease stages (Table 1) as well as responses from PD community members regarding what they wished the doctor had told them about cognitive changes at different stages of their PD (Table 2).

**Table 1:** Questions for working group members

1 What is one recommendation you would give to someone newly diagnosed or early-stage PD:
• To maintain cognitive health?
• Regarding how to cope with cognitive changes?
2. What is one recommendation you would give to someone in mid-stage PD:
• To maintain cognitive health?
• Regarding how to cope with cognitive changes?
3. What is one recommendation you would give to someone with PD as they prepare for cognitive decline associated with advanced PD?
4. What is one recommendation you would give to a care partner regarding how to cope with cognitive changes in their loved one:
• For newly diagnosed or early-stage PD?
• For mid-stage PD?
5. What is one recommendation you would give to a care partner who is taking care of a loved one with advanced PD and experiencing cognitive decline?
6. What do you think healthcare professionals should recommend to patients newly diagnosed or early-stage PD:
• To maintain cognitive health?
• Regarding cognitive changes they may experience?
7. What do you think healthcare professionals should recommend to patients in mid-stage PD:
• To maintain cognitive health?
• Regarding cognitive changes they may experience?
8. What do you think healthcare professionals should tell patients in preparation for cognitive decline associated with advanced PD?
9. What recommendation would you give to academic researchers who may be working with PD patients experiencing cognitive decline?
10. Is there anything else you would want to recommend to either PD patients, care partners, or healthcare professionals? Please tell us here

**Table 2:** Questions for people with Parkinson’s and their care partners

1. In your first year of having PD, what is one thing you wish your doctor told you about:
• Cognitive changes you or your loved one may experience?
• Coping with cognitive changes you or your loved one may have?
2. After having PD for several years, what is one thing you wish your doctor told you about:
• Cognitive changes you or your loved one may experience?
• Coping with cognitive changes you or your loved one may have?
3. What is one thing you wish you doctor would tell you in preparation for cognitive changes in yourself or your loved one associated with advanced PD?
4. Is there anything else you would want to know about cognitive changes, or how to cope with cognitive changes? Please tell us here

In this review, we first provide an overview addressing what is currently known about cognitive dysfunction in PD, describing unmet needs and gaps, and highlighting future directions for each topic covered. Second, based on the conference discussions, we present the working group’s proposed recommendations for people with PD and their care partners when experiencing cognitive issues and for healthcare professionals when working with people with PD facing cognitive challenges.

**Table 3:** Recommendations for maintaining cognitive health across stages of Parkinson’s disease

*Early-stage to mid-stage Parkinson’s disease:*
1. Exercise according to guidelines from American College of Sports Medicine and American Heart Association^60,67,69,71^
2. Stay active socially; for example, spend time with friends or join a support group
3. Engage in cognitive training exercises^81,83,120^
4. Learn coping strategies; for example, work with an occupational therapist or neuropsychologist on techniques for paying attention, remembering things, or doing everyday tasks
5. Nutrition can affect cognition; for example, try a Mediterranean diet
6. Take your time when doing tasks
7. Let your family and friends know if you are having trouble
8. Seek help if feeling depressed or anxious^120^
*Advanced Parkinson’s disease:*
Keep following the above recommendations, *PLUS*:
1. Develop a highly structured daily routine that you follow
2. Consider the use of medication for cognitive impairment
3. Have an advanced directive in place (living will, treatments)^120^
4. For care partners—take care of your own health as well (see doctors as needed)^111^
5. For care partners—seek out support such as counseling

Recommendations based on conference participant feedback and references were noted

Information in this table has been previously published in the blog, Cognitive Problems in Parkinson’s Disease—Beth Vernaleo, PhD at www.movementdisorders.org (March 2017).

**Table 4:** Recommendations for healthcare professionals working with PD patients

1. Provide information at diagnosis regarding cognitive aspects of PD
2. Schedule a follow-up appointment or discussion a few weeks later to answer questions regarding PD
3. Be honest with regard to what changes in cognition can be expected
4. Refer to a neuropsychologist for baseline cognitive testing
5. Have cognition assessed regularly^120^
6. Refer to occupational therapy, physical therapy, speech language therapy, social worker, and nutritionist early and throughout the course of the disease
7. Evaluate patients for depression and anxiety as they can affect cognition^120^
8. Broach the subject of advance planning with patients and caregivers^120^

Recommendations based on PD community conference participant feedback and references were noted

Information in this table has been previously published in the blog, Cognitive Problems in Parkinson’s Disease—Beth Vernaleo, PhD at www.movementdisorders.org (March 2017).

## Current state of knowledge on cognitive impairment in PD

### Characteristics, etiologies, and risk factors

Cognitive impairment in PD is heterogeneous in its severity, rate of progression, and affected cognitive domains.^1^ It varies from subtle cognitive changes or deficits, to mild cognitive impairment (MCI) in which cognitive deficits occur but do not significantly disrupt daily living, to dementia (PDD) in which a greater degree and variety of cognitive deficits substantially affect daily functioning. MCI in PD increases the risk of conversion to dementia.^2,3^ Additional evidence, however, suggests that PD-MCI may not always progress to dementia; on longitudinal assessments, some remain stable as “MCI” and others revert to normal cognition.^2,4,5^ Thus, progression to dementia is not inevitable but develops in about 80% of patients with PD durations, especially longer than 20 years.^6^ Risk factors for PDD include advanced age, older age of disease onset, limited cognitive reserve, hallucinations, and predominant gait dysfunction.^6,7^ Cognitive deficits in PD typically affect executive functions, attention, visuospatial function, and processing speed.^8^ The pattern of cognitive impairment varies, however, in not only the extent to which different cognitive domains are affected but also which domains are affected first. Some people with PD cognitive impairment may have greater memory deficits than non-amnestic dysfunction.^9^ The heterogeneity in presentation and evolution of PD cognitive impairment is not fully understood but may reflect, in part, influences of co-morbid pathologies such as Alzheimer’s disease (AD) or cerebrovascular disease as well as genetics, environment, and cognitive reserve, among others.^10^ The CamPaIGN study provides evidence for the heterogeneity of PD cognitive subtypes and different rates of progression, suggesting a “dual hypothesis”; two distinct cognitive subtypes emerged in this longitudinal study—(1) a frontostriatal/executive function profile associated with dopamine depletion interacting with the COMT genotype and not necessarily progressing to dementia and (2) a posterior cortical dysfunction profile with deficits in impaired language/semantic fluency and visuospatial orientation/pentagon copying, associated with AD pathology, non-dopaminergic transmitters, and apolipoprotein epsilon 4 (*ApoE4*) genotype.^11,12^

Neuropathological studies of patients who died with PDD demonstrate widespread cortical and limbic involvement with neurodegeneration, neuronal loss, and deposition of Lewy bodies and Lewy neurites.^13^ Clinical correlations, however, yield conflicting results as to which neuroanatomical areas and neuropathologies are most important in the clinical expression of PD cognitive impairment.^13^ Basal ganglia pathology, particularly in its associative (cognitive) areas, may also contribute to cognitive deficits.^14^ While PD is an α-synuclein-mediated disease, autopsy studies and cerebrospinal fluid biomarker studies suggest that amyloid pathology contributes to cognitive impairment in PD in some cases.^15,16^ Co-existing synuclein and amyloid pathology may invoke synergistic processes.^17,18^ Cerebrovascular disease contributes to some cases of PD cognitive impairment, with evidence of microvascular ischemia on pathology and white matter hyperintensities on neuroimaging.^13,19^ PD cognitive impairment also reflects dopaminergic, cholinergic, serotonergic, and noradrenergic neurotransmitter deficiencies. Functional neuroimaging and neuropathological studies measuring neurotransmitters support the roles of dopaminergic and cholinergic deficits in PD cognitive impairment.^10,20^

The prevalence of dementia in PD increases with age, disease duration, motor severity, postural instability/gait disorder phenotype, baseline cognitive impairment, and presence of other non-motor and neuropsychiatric issues.^7,21^ REM sleep behavior disorder is closely related to PD cognitive impairment,^22,23^ and greater daytime sleepiness has been associated with worse cognition in PD.^24,25^ Social isolation, depression, and medical illness may worsen cognition in general and in PD. Even after accounting for these factors, however, cognitive function varies among individuals. This variable expression implies potential genetic or environmental modifiers. Some genetic causes or risk factors for PD (e.g., LRRK2) are generally not associated with prominent cognitive dysfunction, whereas α-synuclein duplication and triplications, GBA, and MAPT mutations have been linked to cognitive deficits and dementia.^26–29^ The *ApoE4* allele has been associated with memory and semantic fluency in PD^26,27^ and may increase the risk of PDD, though studies are conflicting.^30,31^ Study results have been conflicting regarding the role of polymorphisms in BDNF and COMT genes in PD cognitive impairment.^11,27,32,33^ In non-PD populations, co-morbidities such as obesity, diabetes, and hypertension may be associated with cognitive decline.^34,35^ This is also the case for diets high in saturated fat, trans-fat, and refined carbohydrates, or low in berries, green leafy vegetables, nuts, vitamin B12, and folate.^36^ At present, data are limited regarding the role of co-morbidities and diet in PD cognitive impairment.^37,38^ Preliminary reports suggest that elevated levels of homocysteine and plasma phospholipids and lower levels of serum uric acid may be associated with worse cognition in PD.^39–41^ Environmental risk factors present compelling opportunities for intervention. However, whether modifying these risk factors would change the progression of PD cognitive impairment is unknown.

## Cognitive change

### What is normal cognitive aging and how does PD fit in?

Many people attribute cognitive changes to “aging,” and a major concern expressed by people with PD and their care partners is whether cognitive deficits are related to aging or to PD. Cognitive changes in people with PD need to be benchmarked against normative data and age-related changes. Cognitive decline without dementia can occur in aging,^42^ perhaps because neuropathological processes such as neuronal loss, deposition of amyloid, tau, and α-synuclein, and vascular changes, often found post-mortem, are common as we age.^43^ The progression of cognitive decline is a key element in attributing changes to underlying disease-related processes. In general, cognitive changes in “normal” aging should not interfere significantly with everyday activities that require cognitive abilities. If they do, however, this may suggest an abnormal process and signal an increased risk of developing MCI or dementia.^44^ Changes in functional abilities and everyday activities due to cognitive decline can be difficult to identify if they are mild. Distinguishing whether problems in everyday activities are due to cognitive or motor problems in PD, or a combination of both, can be challenging, and appropriate measures for determining this are needed.

In “normal” aging, cognitive problems typically involve difficulty with recalling and generating words or names (tip of the tongue phenomenon). Deficits in word or name recall, however, are also common in PD.^1,7^ When objective evidence accompanies subjective cognitive changes without a substantial impact on function, this is defined as MCI,^44^ a concept also applied in PD.^45^ MCI is a risk factor for dementia in both aging^44^ and in PD populations.^44,46^ Community-based studies demonstrate that aging is associated with changes in several cognitive domains, notably speeded measures and recall (aspects of the so-called fluid intelligence), but with relative preservation of others such as vocabulary (crystallized intelligence).^47^ Normative ranges for cognitive performance have been defined for older adults and throughout the life span.

Older people show greater variability on cognitive test performance, particularly in speeded measures (e.g., reaction time), and decline in measures of attention (e.g., divided attention and working memory).^48^ Cognitive fluctuations, impaired attention, slowing, and performance variability are hallmarks of Lewy body disorders including PDD, where they are exaggerated compared to changes of normal aging.^49^ Disentangling effects of age-related and disease-related changes on cognition, co-morbid non-motor issues (e.g., sleep disturbances, mood disorders, apathy, and fatigue), and medications or deep brain stimulation treatments in PD cognitive impairment represent several unmet needs.

## Clinical measures of cognition in PD

Determining objective changes in cognitive function frequently involves neuropsychological testing. Test selection may depend on a number of factors such as age, education, and language. Test performance can be affected by motor function as well as physiological changes of aging (e.g., vision loss, hearing loss, sensory-motor changes). Therefore, neuropsychological testing in PD should include some measures that do not rely on manual dexterity or motor speed as these can be compromised and make it difficult to separate motor symptomatology from a given cognitive ability.

Cognitive function in PD is frequently assessed in clinical and research settings and by physicians, neuropsychologists, occupational therapists, or speech therapists. Evaluations may include tests of global cognitive abilities or individual neuropsychological tests capturing different cognitive domains, or a combination of both.^50^ Clinical measures target the major cognitive domains: orientation, attention, executive function, abstract reasoning, memory, language, perception, visuospatial abilities, praxis, and motor skills.^51^ Methods of cognitive evaluation depend on the goals of the assessment, but formal neuropsychological testing remains a gold standard. It is also important to assess mood (e.g., depression, anxiety, apathy), behavioral issues (e.g., psychosis), sleep disturbances (e.g., excessive daytime sleepiness), and functional abilities in PD as these can impact cognition as well as quality of life. Eliciting cognitive complaints may come directly from the patient, but gathering information from a reliable informant is imperative. The presence of cognitive decline can be gleaned from patient and informant reports, estimates based on premorbid IQ, and serial neuropsychological evaluations. Interpreting studies of cognitive function in PD requires an understanding of not only what is “normal” vs. “pathological” aging and how it is measured but also the context of PD-related motor and non-motor features, effects of medical or surgical treatments, and co-morbid neuropsychiatric complications.

Many tests of global cognition have been studied or reviewed in PD, including the Mini-Mental State Examination, Montreal Cognitive Assessment (MoCA), PD-Cognitive Rating Scale, Addenbrooke’s Cognitive Examination, among others.^45,50,52,53^ Many tests covering different cognitive domains (e.g., attention, executive function, language, memory, or visuospatial functions) also have been used in PD. The Movement Disorder Society (MDS) Task Forces for PDD and PD-MCI diagnostic criteria provide recommendations for assessments of cognitive domains and neuropsychological tests.^45,54^ Standardized protocols and PD-specific neuropsychological batteries may provide uniformity across multiple centers and research trials.

Several other gaps remain in our ability to assess cognition in PD. There is no standard way to elicit cognitive complaints, nor any guidance on which questions are the most sensitive. Cognitive screening tests are frequently not specific for PD cognitive deficits, and their responsiveness to change over time or with intervention is not fully known. The lack of consensus regarding PD cognitive evaluation and defining impairment may contribute to the heterogeneity and potentially false cognitive classifications of people with PD. Normative data and sources vary, and there is little normative data available for those with low education or socioeconomic status. Furthermore, functional assessments lack standardization, although there are several PD-specific tools available including performance-based measures.^55–58^ Validation of the MDS Task Force diagnostic criteria for PD-MCI is underway,^45,59^ and PD-MCI is now being used in clinical trial inclusion criteria. Despite increased recognition of cognitive impairment in PD, cognition is not routinely or consistently assessed in the clinic, in contrast to motor function. Baseline and serial cognitive evaluations need to be considered in order to fully elucidate the full spectrum of cognitive impairment in PD.

## Evidence for non-pharmacological interventions for PD cognitive impairment

### Physical exercise programs: can they help cognition in PD?

Studies in healthy older adults suggest that physical exercise improves cognitive function. A meta-analysis of 29 randomized controlled trials including 2049 participants revealed statistically significant, but clinically modest, improvements in attention, processing speed, executive function, and memory following 6 weeks to 18 months of aerobic exercise (most often performed three times weekly at 70% peak oxygen uptake for 2.5–4 months).^60^ In older adults at risk for cognitive decline, significant improvements in global cognition occurred with at least 150 min of moderate-intensity physical activity per week over 6 months, with benefits persisting after 18 months.^61^ However, among sedentary older adults in the Lifestyle Interventions and Independence for Elders (LIFE) study, a 24-month moderate-intensity exercise program (aerobic, resistance, flexibility exercises) compared to a health education program did not improve global or domain-specific cognitive function. Participants in the physical activity group who were 80 years or older or had worse baseline physical performance, however, had greater changes in executive function composite scores compared to the health education group.^62^

Studies examining the effects of physical exercise on cognitive function in PD are emerging, and results are promising.^63–66^ Aerobic exercise may improve executive function in PD,^67^ consistent with findings in studies of healthy older adults. In one study, 49 people with mild to moderate PD completed participation of 45 min of aerobic walking three times weekly over 6 months; there were significant improvements in exercisers in executive function (Flanker task scores) along with improvements in scores for motor severity, fatigue, depression, and quality of life.^68^ In a randomized controlled trial (*n* = 28), people with PD improved specifically in executive function measured by verbal fluency and spatial working memory tasks following participation in 60 min of progressive aerobic and anaerobic training twice weekly over 3 months.^69^

Strengthening exercises also may benefit cognition in PD. In a randomized controlled trial, 51 people with mild to moderate PD participated in either a progressive resistance exercise program or a non-progressive strengthening program twice weekly over 2 years. Participants in both groups improved in working memory (Digit Span) and executive function (Stroop test) at 24 months, whereas only the progressive resistance group had significantly better attention.^70^ In a multi-modal exercise study (aerobic, strengthening, flexibility, and balance exercises) involving 20 people with PD, significant improvements in executive function (Wisconsin Card Sorting Test) occurred in the exercise group compared to the control group.^71^ A community-based 12-week tango program (90 min, twice weekly) in 24 people with mild to moderate PD demonstrated improved spatial cognition compared to those who participated in educational sessions (*n* = 9).^72^ Collectively, these results provide preliminary evidence for benefits of different types of physical exercise on cognitive function, particularly executive functions, in people with mild to moderate PD.

Several questions remain unanswered regarding effects of physical exercise on PD cognitive impairment or decline. We need to know more about the optimal type(s) of exercise (i.e., aerobic, strengthening, or a combination), dose (e.g., frequency, intensity), duration, and timing to improve cognitive function in PD. We need a better understanding of the neurobiological and physiological mechanisms of physical exercise (i.e., modulation of dopamine transmission, neurotrophic factors, corticomotor excitability, and structural, functional, and metabolic changes in the brain, among others) that may account for cognitive improvements observed. We need to understand if the impact of physical exercise on cognition is global or selective for particular cognitive domains (e.g., executive function, memory). These factors will shape clinical trial design in the selection of target outcome measures and optimal PD populations and disease stages. As many studies to date have small sample sizes, did not include control groups, and used various interventions and doses, rigorously designed trials are needed. Cognition needs to be measured as the primary outcome, which is not always the case in previous studies of exercise and cognition in PD. An understanding of the impact of physical exercise on cognitive function during real-world activities is needed.

### Cognitive interventions: can they help cognition in PD?

There are several different types of cognitive interventions. Cognitive remediation or rehabilitation techniques include broad, multi-component approaches typically aimed at recovering an individual’s abilities in the face of loss (e.g., stroke or head injury). Some approaches may be indirect (i.e., targeting a third variable such as mood or social/activity engagement that may thereby influence a cognitive process or strategy),^73–75^ and others involve multifaceted therapeutic techniques such as combining cognitive training, support groups, wellness education, and/or mental health counseling.^76^ Cognitive training refers to specific “brain exercises” that involve targeted practice with the goal of improving particular cognitive abilities (e.g., memory, speed of processing, attention, executive function) and by using different cognitive training techniques or strategies (e.g., paying attention to, remembering, planning, or organizing information). Cognitive training is based on the idea that cognitive functions can be “strengthened” over time with practice, similar to physical exercise strengthening muscles. Non-pharmacological approaches like cognitive training are of great interest, due to their potential to alleviate cognitive symptoms, provide individualized approaches, and avoid medication side effects. However, there has been rising public concern that many programs and products claim to stave off cognitive decline and dementia. Commissioned experts evaluated the science behind these interventions and noted that cognitive training was one of the few interventions (along with physical exercise and blood pressure control) to receive a positive recommendation and “encouraging” evidence from the National Academies of Sciences, Engineering, and Medicine committee.^77^

Several studies examine the effects of cognitive interventions in PD, but few provide conclusive evidence regarding benefits of engaging in such activities.^78^ To date, no cognitive intervention studies among persons with PD meet all of the criteria proposed in the Institute of Medicine’s Report on Cognitive Aging for evaluating cognitive training programs.^79^ These criteria include: reliable transfer to other similarly constructed laboratory training tasks, transferability to relevant real-world tasks, efficacy established through comparisons of active control and experimental groups with equivalent expectations for benefit, skill retention post training, and replicability across studies. Nonetheless, preliminary evidence holds promise. Data from the few well-designed randomized controlled trials of cognitive training in PD conducted to date reveal large and statistically significant effect sizes for cognitive domains known to be impaired in PD, such as working memory, processing speed, and executive functioning (i.e., also seemingly more frontostriatal-type deficits), but not posterior cortically based cognitive tasks.^78,80–86^ Cognitive interventions (e.g., cognitive-behavioral therapy), which target common non-motor symptoms such as depression and anxiety also may provide improvements in select cognitive domains (e.g., working memory, executive skills).^75^

Future research must address several key questions including what specific training tasks positively impact real-world cognitive abilities, what factors influence training outcomes, which cognitive deficits in PD improve and whether this affects risk of cognitive decline or dementia, whether cognitive improvements in one area transfer to another, and what neurobiological mechanisms underlie effects of cognitive interventions. Multi-component programs targeting specific cognitive deficits may confer the greatest benefit but require additional study. Large, long-term, randomized controlled trials enrolling people with PD at all disease stages, from early, or even at risk, to advanced stages, are needed to determine whether specific types of cognitive training can prevent, delay, or mitigate cognitive and functional decline. Incorporating patient-centered outcomes will ensure that we are meeting patient needs with regard to which cognitive functions are most important for daily life. Apathy and fatigue, which can be major impediments to participation in physical or cognitive exercises or social activities for some patients with PD, also will be important areas to target.

### Nutritional interventions: can they help cognition in PD?

Good nutrition is essential for living well in general and with PD, not only playing a role in optimizing general health and motor strength but also potentially cognitive function. The relationship between nutrition and cognition is an area of growing interest to the PD community. In a large cross-sectional survey conducted by the Parkinson Alliance (*n* = 1492), 93% of participants reported that they believed that diet/nutrition was important in managing their PD symptoms.^87^ Yet, only 11% of participants reported that a healthcare professional offered specific dietary recommendations to them. The majority of participants who followed a specific diet “designed” their meal plans based on information obtained from self-help resources (e.g., internet, magazines), family, and friends. Moreover, while 63% of respondents perceived themselves as eating a “healthy” diet most of the time, there is a lack of consensus as to what comprises a healthy diet for people with PD. Discussions regarding nutrition in PD have typically focused on topics such as protein and medication absorption, weight loss, dysphagia, and gastrointestinal issues. However, there are growing investigations of nutrients that may be associated with increased or decreased risk of PD. Increased consumption of dairy products^88^ and lower serum urate levels may be risk factors for PD,^89^ whereas high intake of fruits, vegetables, and fish or use of nicotine and caffeine may confer a lower risk of PD.^90,91^

Research on the relationship between nutrition and cognition in general and in PD populations is increasing. Studies in non-PD populations suggest that foods such as blueberries, cocoa, dark chocolate, tea, and wine may protect against cognitive decline (e.g., global cognition, verbal memory, executive skills, and verbal fluency) over time.^92–94^ Specific dietary patterns (e.g., Mediterranean, DASH, and Mediterranean-DASH Intervention for Neurodegenerative Delay (MIND)) may provide healthful effects in non-PD groups.^95–98^ In one study, the MIND diet was associated with a 54% reduction in risk for AD. In a longitudinal cohort study, adoption of more “MIND-like” dietary patterns was associated with less decline in global cognition, processing speed, and executive function over a 4.7-year period, compared to “traditional” patterns of eating.^95^ Nutritional supplements (e.g., folate, vitamin B12) may confer cognitive benefit, but dosages must be carefully considered.^99,100^ Studies of nutritional interventions need to consider the ways to best facilitate dietary behavior change and how to best monitor dietary behaviors, food intake, and dietary supplements. In addition, it will be important to examine potential confounders such as psychosocial factors, concomitant exercise/physical activity, presence of co-morbid medical or metabolic illnesses, and genetic variants (e.g., *ApoE*) as well as methodological issues such as the level of benefit from individual nutrients and/or foods and use and interpretation of dietary pattern scores.^101^

To date, few studies have assessed interactions between nutrition and cognition specifically in PD. A meta-analysis suggests that higher homocysteine levels and lower folate and vitamin B12 levels were related to cognitive dysfunction in PD,^102^ though the relationship of elevated homocysteine and PD cognitive impairment is not fully established. Low plasma uric acid predicted worse performance on cognitive tests in cross-sectional and follow-up studies in PD.^103^

Higher vitamin D concentrations have been associated with better performance on tests of verbal fluency and verbal memory in non-demented PD patients, but this requires further study.^104^

Randomized controlled trials and longitudinal studies are needed to determine the effect of individual food compounds, supplements, and dietary patterns on cognitive functioning and cognitive decline in PD. We need evidence-based nutritional recommendations for enhancing cognitive function for people with PD. We will need to distinguish between symptomatic and potentially disease-modifying effects of nutritional choices. Furthermore, we will need to define the optimal timing and dosage of any dietary changes and who may benefit most from nutritional interventions. Effective behavioral strategies for facilitating healthy nutritional change will also be important.

## Psychosocial considerations regarding cognitive decline in PD

### Care partners: burden, assessment, and support

Care partners or caregivers are an invaluable healthcare resource. When caregivers are present, patients are less likely to move to nursing homes.^105^ Caregivers also improve participation rates and retention of people with PD in research studies, which helps advance the path towards finding more effective therapeutics.^106^ Furthermore, many clinical trials for PDD require a caregiver in the study inclusion criteria.

Informal caregivers are providers who supervise or assist with instrumental and/or basic activities of daily living without pay to someone who cannot do these activities independently due to cognitive, physical, or psychological impairment. However, caregivers provide much more than this formal definition states. They often provide medical care including administration of medications, emotional and social support, and advice on medical decision-making. About 90% of men and 80% of women with PD have caregivers during their physician visits,^107^ and most caregivers are spouses.

Unfortunately, caring for a person with PD can lead to caregiver burden and strain. When a person with PD experiences greater cognitive impairment including dementia, neuropsychiatric symptoms, and motor difficulties, health-related quality of life declines and caregiver burden increases.^108–110^ Caregiver strain has been linked to higher mortality.^111^ While there is strong evidence that caregiving can be burdensome and negatively affect the caregiver’s health and well-being, caregiving also can have positive and rewarding aspects (e.g., taking pleasure in giving, feeling purposeful, bringing people closer). Comprehensive, multidimensional assessment of caregiver needs and strains are vital in developing effective, targeted plans to address their needs.^112,113^ Some assessments that can be used to gauge caregiver needs and degree of burden include: interRAI home care assessment, American Medical Association Caregiver self-assessment questionnaire, Zarit Burden Interview, and Multidimensional Caregiver Strain Index.

Current strategies to support caregivers center around three main areas: education, psychosocial support, and psychotherapeutic interventions. Education should start early at patient diagnosis and maintain a “patient-centered,” tailored approach. Educational topics include PD symptoms and progression, medication management, care coordination, resources, communication with the healthcare team, and self-management strategies. Psychosocial support provides an opportunity to discuss the caregiving role with the patient and family. This can be achieved via mental health providers (e.g., LICSW, psychologist) or PD support groups. Specific psychotherapeutic interventions such as Cognitive Behavioral Therapy, Relaxation Therapy, and Strength Perspectives Therapy can aid the caregiver’s well-being. A multidisciplinary team approach (e.g., physician, nurses, therapists, social workers) helps to identify at-risk caregivers and provide appropriate education, support, and referrals.

Despite existing therapeutic support and resources for caregivers, there are several challenges to overcome. These include sex disparities in caregiving (e.g., women with PD are less likely to have informal caregivers then men) and the substantial financial strain that can be associated with caregiving. Caregivers are frequently at the forefront of providing medical history and administering complicated medication regimens without formalized education or support.

Future research should include developing ways to prevent or lessen caregiver strain. Comparative-effectiveness research of different education, skill-building and support resources for caregivers of people with PD experiencing cognitive impairment may be necessary to help allocate resources. Assessments of current caregiver needs are necessary to inform health policy for people with PD especially with cognitive impairment.

### Decisional capacity considerations

Establishing decisional capacity is an important consideration in the setting of cognitive impairment. Intact decisional capacity includes the abilities to: understand relevant information, appreciate the situation and its likely consequences, manipulate information rationally (reasoning), and have evidence of a choice.^114^ Capacity is important for consenting for medical/surgical procedures, living independently, and making financial decisions. Defining capacity is task-specific and situation-specific and can be temporary and reversible. Investigations of capacity to consent for research participation show that people with PD with mildly impaired cognition have difficulty understanding medical scenarios when compared to cognitively normal PD.^115^ With greater cognitive impairment, appreciation and reasoning are also affected. Specific cognitive domains that influence reasoning capacity in PD include executive function and memory.^116^

Since even having MCI may affect capacity, it is important to assess decision-making ability in both clinical and research settings. Global cognitive tests such as the MoCA can provide an index of the likelihood of impaired capacity. There also exist tools specifically for capacity assessment including the MacArthur Competence Assessment Tool for Treatment (MacCAT-T) and Aid to Capacity Evaluation (ACE). Capacity assessments should include inquiry about co-morbid medical conditions (e.g., hearing loss), medications that could contribute to apparent diminished capacity, physical and cognitive functional assessments, and environmental evaluations.

Altered decisional capacity in PD is under-recognized. It can be difficult to detect in the context of established routines and may not present itself until there is a novel demand or unfamiliar task. However, performance on the MoCA in PD correlates with capacity to understand discussions about goals of care.^117^ Training healthcare professionals to recognize impaired decisional capacity, to conduct an careful assessment, and to interpret the findings is essential, as well as having a planned course of action once it is clear that capacity is impaired. Occupational therapy can be useful for evaluating patient abilities and safety at home and when driving as well as for teaching skills to improve performance and quality of life.

Current challenges include limited resources and training available regarding capacity assessments for healthcare professionals, research staff, and people with PD and their care partners. There are no clear guidelines on how to systematically approach decision-making capacity in PD (e.g., what is the role of clinical judgment, at what time intervals should capacity be re-assessed).

Future research directions in PD include examining the consequences of executive dysfunction on global decision-making and changes in decision-making capacity over time. Lastly, understanding the implications of capacity cut-off scores are important for clinical trials participation, ethical and regulatory oversight, and legal assessment.

## Questions and recommendations

After discussion of the nine topics presented in this review, conference participants were tasked with producing questions to ascertain what recommendations they would give, as healthcare providers, to a person at early, mid, or advanced PD stages regarding maintaining their cognitive health and coping with cognitive changes (Table 1). This feedback then generated the recommendations listed by attendees, which include non-pharmacological strategies (physical and cognitive exercise), mental health wellness, psychosocial coping skills, and planning (Table 3). These recommendations include several with a strong evidence base (e.g., guidelines from the American College of Sports Medicine and the American Heart Association for physical exercise) and others with some literature support but requiring further study and for which several are currently under study (e.g., cognitive training exercises, Mediterranean diet). The feedback from attendees also included anecdotal, and often “common sense,” recommendations such as staying active socially, learning coping strategies, taking ones time when doing tasks, seeking help if feeling depressed or anxious, or taking care of the care partner too. Overall, these recommendations can be incorporated into the clinical care for people with PD and their care partners. Exercise, diet, and cognitive training interventions should be discussed with one’s healthcare providers for the best way to implement them, and related health specialists such as physical therapists, nutritionists, and neuropsychologists should be consulted and part of the care team. Furthermore, future research on outcomes of their utilization will help elucidate their impact and effect.

From the group discussions, questions were developed by the people with PD and their care partners focusing on what they wished they had known from their healthcare provider regarding cognitive changes (Table 2). These responses provide a unique opportunity to have greater insight into what people with PD and their care partners hope to achieve in their relationships with their healthcare provider, throughout the entire course of PD. The views of people with PD and their care partners suggest that they have a desire for information at diagnosis regarding cognitive aspects of PD, a topic that may not be readily discussed, as well as an interest in being proactive about non-pharmacological strategies for PD across all stages. In addition, the group recommendations, as endorsed by people living with PD, include regular follow-up with healthcare professionals for discussions about PD symptoms, cognitive and mood assessments, and future planning and referrals to allied therapists early and throughout the PD course (Table 4). Direct input from people living with and affected by PD offers an opportunity for the healthcare providers to hear what matters to patients and their care partners, change the way that care is provided from the moment of diagnosis, and empower people with PD and their care partners to be actively engaged and proactive with their care.

## Current/future research

At present, there is only one Food Drug Administration-approved medication for PDD in the United States, that is, the cholinesterase inhibitor, rivastigmine, which was approved in 2006. Evidence-based reviews in PD recommend rivastigmine as “efficacious” for the treatment of PDD, with an acceptable safety risk without need for specialized monitoring.^118^ Other cholinesterase inhibitors, donepezil (despite a large randomized controlled trial)^119^ and galantamine, and memantine were rated as having “insufficient evidence” in the treatment of PDD, though they had acceptable safety risk profiles without need for specialized monitoring.^118^ Other agents, particularly focused on the serotonin system, are currently under study for PD dementia.^65^ Cholinesterase inhibitors, MAO-B inhibitors, and stimulant-like medications have been studied in PD-MCI, but trials have been limited by small sample sizes, lack of uniform definitions, use of different outcome measures, and negative results. Given the overall lack of pharmacological therapies for PD cognitive impairment, non-pharmacological therapies are of great interest. Studies of non-pharmacological therapies such as physical exercise cognitive training, and neuromodulation in PD cognitive dysfunction are growing, though more rigorous study is needed. Recently, the American Academy of Neurology updated the 2001 practice guidelines for the diagnosis and management of MCI; while this publication focused on MCI in AD, many of these recommendations may extend to MCI in PD.^120^

In order to advance treatments for PD cognitive impairment and promote ways to maintain cognitive health throughout disease, several key issues need to be addressed. We will need to identify strategies and therapies that address the full spectrum of PD cognitive impairment with its heterogeneity of phenotype and progression. We will need to develop and utilize statistical models and predictive algorithms such as ones recently devised that incorporate clinical features and genetics into scores for individualized risk of subsequent dementia.^121^ We will need to identify predictive biomarkers of cognitive impairment that can be readily implemented in research and clinical settings. We will need to develop and optimized measures for screening cognition in PD and for use as primary and secondary outcomes in clinical research trials. We will need a clear regulatory pathway for pharmacological and non-pharmacological interventions for PD cognitive impairment, including the PD-MCI stage, and for not only symptomatic, but also disease-modifying agents that prevent or slow cognitive decline.^122^ As these therapies come to fruition, we will need to have appropriate insurance coverage for them. Lastly, we will need better support and education regarding cognitive changes for people with PD and their care partners, early on in the disease as well as throughout their disease course.

### Data availability

Data sharing is not applicable to this article as no datasets were generated in this study.
